# Low 30-day mortality and low carbapenem-resistance in a decade of *Acinetobacter* bacteraemia in South Sweden

**DOI:** 10.1080/20008686.2021.2009324

**Published:** 2021-12-10

**Authors:** Erik Ingefors, Jonas Tverring, Fatima Nafaa, Niklas Jönsson, Sara Karlsson Söbirk, Charlott Kjölvmark, Oskar Ljungquist

**Affiliations:** a Department of Infectious Diseases, Helsingborg Hospital, Helsingborg, Sweden; b Division of Infection Medicine, Department of Clinical Sciences, Lund University, Lund, Sweden; c Clinical Microbiology, Infection Prevention and Control, Region Skåne, Lund, Sweden; d Clinical Infection Medicine, Department of Translational Medicine, Faculty of Medicine, Lund University, Malmö, Sweden

**Keywords:** Antimicrobial resistance, opportunistic infections, blood stream infections, MALDI-TOF, nosocomial infection

## Abstract

**Background:**

The aim of this study was to provide a descriptive account of carbapenem resistance and risk factors for mortality from invasive *Acinetobacter* infections in the south of Sweden.

**Methods:**

Blood isolates with growth of *Acinetobacter* species between 2010 and 2019 in Skåne county were subtyped using MALDI-TOF and subjected to susceptibility testing against clinically relevant antibiotics. Association between risk factors and 30-day mortality were analysed in univariate and multivariate logistic regression models.

**Results:**

There were 179 bacteraemia episodes in 176 patients included in the study. The 30-day all-cause mortality was 16%. In all, two percent of *Acinetobacter* strains were carbapenem resistant. Independent risk factors associated with 30-day mortality in the multivariate regression model were *Acinetobacter* growth in all blood cultures drawn at the day of bacteraemia onset (OR 5.0, 95% CI: 1.8 to 13.7, *p*= 0.002), baseline functional capacity (1–4 points, OR 2.0, 95% CI: 1.2 to 3.4, *p*= 0.010) and correct empiric antibiotics at time of culture (OR 3.5 95% CI: 1.0 to 11.8, *p*= 0.045).

**Conclusion:**

This study on *Acinetobacter* bacteraemia in South Sweden found low 30-day mortality and low carbapenem-resistance rates compared to previous international studies which may be due to a higher rate of contaminant findings.

## Introduction

The bacterial genus of *Acinetobacter* is a diverse group comprising gram-negative, strictly aerobic coccobacilli of more than 60 different species [1,2]. *Acinetobacter* is primarily known as an opportunistic pathogen causing nosocomial infections, particularly in intensive care units (ICUs). Risk factors for infection include longer duration of hospitalization, exposure to broad-spectrum antibiotics, mechanical ventilation, venous and urinary catheterization and other impairments of natural barriers such as wounds and surgical procedures [3,4]. Pneumonia (commonly ventilator-associated) and venous catheter-related bacteraemia dominate the infection-type spectrum, but urinary tract infections, wound infections and endocarditis, amongst others, are also frequently reported [3,4]. Community-acquired *Acinetobacter* infections occur, albeit less frequent compared to nosocomial infections [3,4]. *A. baumannii* is the most common species to cause infection[3].

The southern and eastern parts of Europe saw the highest rates of carbapenem resistance amongst invasive *Acinetobacter* isolates in Europe in 2019, with Greece, Croatia and Romania reporting rates of 92%, 92% and 88%, respectively[5]. *A. baumannii* strains resistant to all available antibiotics have been reported[6].

Even though *Acinetobacter* is a pathogen of increasing importance globally, data on *Acinetobacter* infections in Sweden are scarce. Contrary to other bacteria with large potential for antibiotic resistance, *Acinetobacter* is not under national surveillance. We aimed to investigate invasive *Acinetobacter* infections in our clinical setting in the south of Sweden. The study objective was to provide a detailed descriptive account of invasive *Acinetobacter* infections with regard to epidemiology, antibiotic resistance, clinical characteristics, treatment and outcome.

## Materials and methods

### Study design and data collection

We performed a population-based observational study in Skåne County in southern Sweden, with a population of 1.3 million[7]. Data on blood cultures positive for *Acinetobacter* species (spp.) between the years of 2010–2019, including personal identification numbers, were obtained from the Department of Clinical Microbiology at Skåne University Hospital. This department handles and analyses all microbiological cultures from primary, secondary and tertiary care in Skåne. All blood cultures positive for an *Acinetobacter*-species during the study period were included. Exclusion criteria was complete absence of data in medical records and clinical microbiology records. Medical records were reviewed using the software Melior (Melior, Siemens Healthcare Services, Upplands Väsby, Sweden). Laboratory Registers at the Department of Clinical Microbiology, Region Skåne, were accessed through LIMS-systems ww-lab (Autonik, Nyköping, Sweden) and Bactius (Clinical Microbiology, Lund, Sweden).

### Definitions of variables

Multiple blood cultures positive for *Acinetobacter* during the same hospitalization were considered one single event of bacteraemia. The bacteraemia was registered as carbapenem resistant if at least one of the blood cultures with *Acinetobacter* growth were carbapenem resistant. Patients with repeated bacteraemia at different episodes of in-patient care could be included multiple times. Cases were classified as primary bacteraemia if no other infectious foci were found and secondary if another focus was present (ie presence of positive *Acinetobacter* cultures from other locations, as well as the clinical assessments of the treating physician). Bacteraemia were defined as nosocomial if the first blood cultures positive for *Acinetobacter* was drawn after more than 48 hours of hospitalization, otherwise the infection was defined as community acquired. The variable ‘abroad recently’ was defined as abroad stay within 30 days prior to the bacteraemia onset. Immunosuppression was defined as a daily corticosteroid dose equivalent to ≥15 mg of prednisolone, chemotherapy or other medication with significant immunosuppressive effect, such as TNF-alpha inhibitors, or innate immune deficiencies. Charlson comorbidity index was calculated according to the model developed and validated by Charlson et al [8]. National Early Warning Score 2 (NEWS2) was calculated in accordance with the model developed by Royal College of Physicians[9]. A modified version of the Brighton Paediatric Early Warning Score (PEWS) proposed by Solevåg et al was used for patients aged <18 years [10]. Baseline functional capacity was defined according to the categories used by Dickstein et al [11].

### Microbiological analyses

All blood isolates drawn between 2010 and 2019 and preserved at the Department of Clinical Microbiology at Skåne University Hospital Lund with growth of *Acinetobacter* spp. were re-cultured. Isolates were plated on Mueller-Hinton-agars (Oxoid, Basingstoke, UK) and incubated for 18 hours. Isolates were tested for susceptibility to cefiderocol, doripenem, imipenem, meropenem, imipenem-relebactam, ciprofloxacin, levofloxacin, amikacin, gentamicin, tobramycin, colistin and trimethoprim-sulfamethoxazole by disc diffusion using antibiotic-impregnated discs (Oxoid, Basingstoke, UK for all but colistin which were from Mast Group, Bootle, UK) and E-tests (bioMérieux, Askim, Sweden and Liofilchem, Roseto degli Abruzzi, Italy). Inhibition zone diameters were interpreted using the NordicAST (Nordic Committee on Antimicrobial Susceptibility Testing) breakpoints (www.nordicast.org). For strains not preserved (n = 72), the original susceptibility testing (performed at time when the *Acinetobacter* strain was uncovered) was used. *Acinetobacter* strains were identified to species level using Matrix Assisted Laser Desorption/Ionization-Time Of Flight (MALDI-TOF) Mass Spectrometry (MS) and analysed with software MALDI Biotyper (MBT) Compass (Bruker, Billerica, Massachusetts, USA).

### Statistical analyses

We used multivariable logistic regression as our main statistical method for investigating an association between putative risk factors and the outcome. We limited the number of investigated risk factor to six due to a limited sample size and few events. Variables were chosen in two steps, the first part was based on a known or suspected association with the outcome to enable a well calibrated survival model (age, comorbidities, baseline functional capacity and vital parameters). The second part was more exploratory, and variables were chosen to investigate putative associations with the outcome (all blood cultures positive and correct empiric antibiotics). We first fit univariate logistic regression models using all variables on their original scale and using complete case analysis respectively. We investigated patterns of missingness and concluded there to be a missing at random mechanism with >5% missingness in at least one variable. We performed chained multiple imputation using predictive mean matching and 10 imputations including all variables in the final model, the outcome variable, and additional data on nosocomial infection, immunosuppression and ICU admission. Non-linearity between the included variables and the outcome was tested with likelihood ratio tests using 4 knot restricted cubic splines for continuous variables and categorization for factor variables. We then fit a multivariable logistic regression model using original and imputed data and reported odds ratios, 95% confidence intervals and *p-*values for all variables together with measures of discrimination (area under receiver operating curve) and model fit (pseudo R^2^) for the full model. We checked model assumptions using variable inflation factor <4 for multicollinearity and Hosmer-Lemeshow goodness-of-fit test for calibration. All results are reported with 95% confidence intervals and *p-*values below 0.05 were regarded as statistically significant. Statistical analyses were performed using Stata MP version 16.1 (StataCorp, College Station, Texas, USA).

### Ethics

The study received ethical approval. This committee waived the need for written consent from the patients of the study due to the retrospective nature of the study and the fact that the patients are unidentifiable. The study was performed in accordance with the Declaration of Helsinki.

## Results

### The yearly incidence of Acinetobacter bacteraemia did not increase over the study period

Between 2010 and 2019, there were 233 blood cultures positive for *Acinetobacter* species in Skåne. When comparing multiple positive blood cultures against hospitalization episodes, 189 cases of bacteraemia were identified. Of these, 10 cases were excluded from the study due to absence of data. Thus, 179 cases of bacteraemia in 176 patients were included in the study; three patients had two episodes of bacteraemia each.

**Figure 1.**
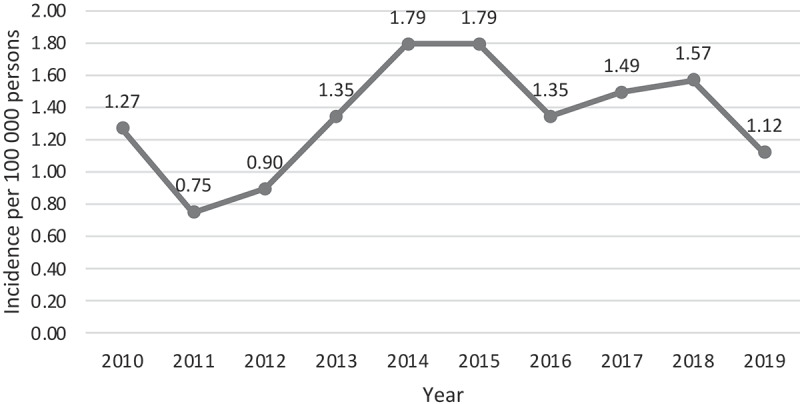
Yearly incidence rate of included blood stream infections with *Acinetobacter*, 2010–2019

The yearly incidence of *Acinetobacter* bacteraemia in Skåne remained fairly constant during the study period, ranging between 0.75 and 1.79 cases per 100,000 citizens and year (mean and median 1.3). Yearly incidence of cases 2010–2019 are displayed in Figure 1.

### Baseline characteristics of the study population

**Figure 2.**
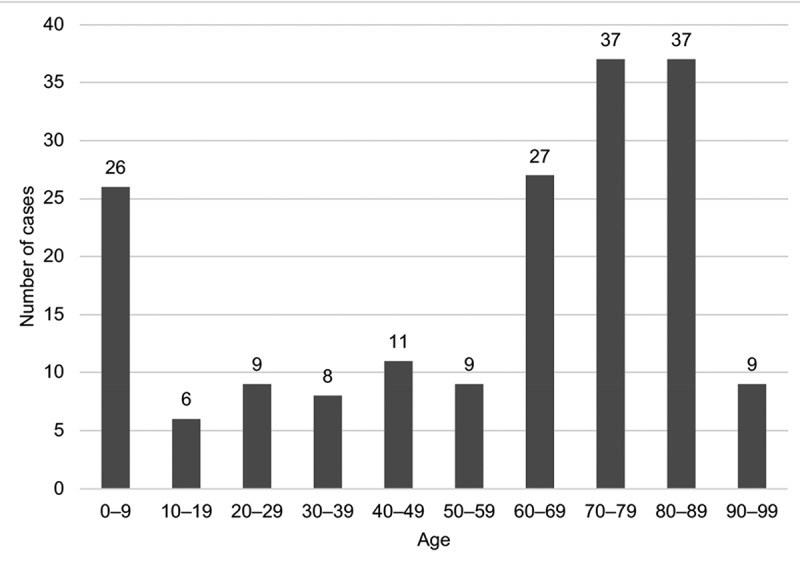
Patient age distribution for the included cases

Out of the 179 included cases of bacteraemia, 92 (51.4%) occurred in male patients, and the median age was 67 years (range 0–94). The median Charlson comorbidity index score was four (range 0–15), and 44 patients (25%) were considered immunosuppressed. The median length of hospitalization was 10 days (range 0–320), and 26 patients (15%) were admitted to the intensive care unit (ICU). The baseline characteristics of the study population are presented in Table 1 and age distribution of patients is displayed in Figure 2.

**Table 1.** Patient characteristics

	*n* total = 179	*n* missing (%)
Male sex, *n* (%)	92 (51.4)	
Age, median years (range)	67 (0–94)	
Charlson comorbidity index, median score (range)	4 (0–15)	
Immunosuppressed patients ^a^, *n* (%)	44 (24.7)	1 (0.6)
Baseline functional capacity, *n* (%)		20 (10)
Fully functional	84 (52.8)	
Functional with minimal assistance	36 (22.6)	
Require assistance with ADL	27 (17,0)	
Bedridden	12 (7.6)	
Admitted from, *n* (%)		
Home	154 (86.0)	
Care home	13 (7.3)	
Other hospital	11 (6.1)	
Institution	1 (0.6)	
Reason for admission, *n* (%)		
Suspected infection ^b^	111 (62)	
Other	66 (37)	
No admission ^c^	2 (1)	
Duration of hospitalization, median days (range)	10 (0–320)	
Treated in ICU, *n* (%)	26 (15)	
Length of ICU stay, median days (range)	6 (1–33)	
Abroad stay prior to hospitalization ^d^, *n* (%)	10 (6)	
Care level abroad, *n*		
No medical care	3	
Outpatient care	0	
Hospital ward	2	
ICU	5	
NEWS2/PEWS at bactaeremia onset, median (range)	5 (0–12)	26 (15)
Biochemical measurements in all ages, median (range)		
Blood lactate, mmol/l	1.8 (0.5–23)	70 (39)
C-reactive protein, mg/l	57 (0.6–482)	22 (12)
Procalcitonin, µg/l	0.84 (0.13–67)	162 (91)
Risk factors for infection, *n* (%)	28 (16)	
Hemodynamic support with vasoactive drugs at onset	5	
NIV at onset	2	
Ventilator treatment at onset	9	
Haemodialysis (IHD or CRRT) at onset	3	
Major surgery within 3 days prior to onset	9	

^a^Immunosuppression was defined as a daily corticosteroid dose equivalent to ≥15 mg of prednisolone, chemotherapy or other medication with significant immunosuppressive effect, such as TNF-alpha inhibitors. Immunosuppressive haematologic disorders were also included in this category

^b^Including suspected airway, urinary tract, skin, neurological and unknown focus

^c^Patient discharged directly from emergency room

^d^Denmark, Greece, Iceland, Kenya, Norway, Poland, Serbia and Syria.

ADL: activities of daily living. ICU: intensive care unit. NEWS2/PEWS: National/Pediatric early warning score. NIV: non-invasive ventilation. IHD: intermittent haemodialysis. CRRT: continuous renal replacement therapy.

### The majority of the episodes of bacteraemia were community-acquired

Out of the 179 episodes of bacteraemia included in this study, 139 (78%) were community-acquired (Table 2). Of the 40 nosocomial cases, the median time to bacteraemia was 15 days (range 2–136). Out of the 26 patients treated in the ICU, 16 (62%) had onset of bacteraemia before ICU admission, while six (23%) had onset during the ICU stay and four (15%) after ICU discharge. Ten patients (6%) had been abroad recently prior to hospitalization. At the time of bacteraemia onset, 47 patients (26%) were treated with antibiotics.

**Table 2.** Bacteraemia and treatment characteristics

		*n* missing (%)
Bacteraemia onset ^a^, *n* (%)		
Community-acquired	139 (78)	
Nosocomial	40 (22)	
Time to nosocomial bacteraemia onset, median days (range)	15 (2–136)	
Bacteraemia onset in ICU patients, *n* (% of ICU treated patients)		
Before ICU admission	16 (62)	
During ICU stay	6 (23)	
After ICU discharge	4 (15)	
Growth of additional bacteria in blood culture, n (%)	81 (46.3)	4 (2)
*Acinetobacter* in culture from other location ^b^, n (%)	20 (12)	6 (3)
Primary/secondary bacteraemia^c^, n (%)		
Primary	82 (46)	
Secondary, suspected focuses below	97 (54)	
Carbapenem resistant *Acinetobacter*, n (%)	5 (2.96)	10 (6)
Ongoing antibiotic treatment at time of bacteraemia onset ^d^, n (%)	47 (26)	
Received empirical antibiotic treatment, n (%)	164 (91.6)	
Received effective antibiotics at bacteraemia onset ^e^, n (%)		2 (1)
Yes	37 (20.9)	
No	140 (79.1)	
Received effective antibiotics after antibiogram was available, n (% of those previously untreated ^f^)	104 (74.2)	
Time to start of effective antibiotics if initially untreated ^f^, median (range)	2 (0–17)	
Received effective antibiotics at any time during the course of bacteraemia ^e^, n (%)		3 (2)
Yes	133 (75.6)	
No	43 (24.4)	
Effective antibiotic treatment time, median days (range)	10 (1–28)	
Received source control treatment ^g^, n (%)	33 (18.4)	
Withdrawal of care or suspected contamination ^h^, n (%)	53 (29.6%)	

^a^Infection was regarded community-acquired (≤48 hours) or nosocomial (>48 hours) based on the time at which the first blood culture positive for *Acinetobacter* was taken

^b^Includes VAP, wound, urine, CVC, gall, nasopharynx, ascites, rectum and perineum

^c^The classification of infections as primary or secondary was based both on the presence of positive *Acinetobacter* cultures from other locations, as well as the treating physicians’ clinical assessments. Suspected focus of secondary bacteraemia include.airways, CNS, CVC, factitial, GI, nephrostomy, PAC, PICC-line, post-operative, skin and urine

^d^Including antibiotic treatment discontinued within one day before bacteraemia onset. ^e^As determined later by antibiogram

^f^Includes both those completely untreated and those ineffectively treated empirically

^g^Included but not limited to catheter removal, nephrostomy insertion and wound revision

^h^Effective treatment discontinued or not at all initiated, both including cases where the patient had too poor prognosis to motivate further treatment and cases where the *Acinetobacter* was deemed a contamination. Bacteraemia onset referrers to when the blood cultures were drawn.

ICU: intensive care unit; VAP: ventilator associated pneumonia; CVC: central venous catheter; GI: gastrointestinal; PAC: port-a-cath; CNS: central nervous system, UTI: urinary tract infection;

### Few strains were carbapenem-resistant

**Figure 3.**
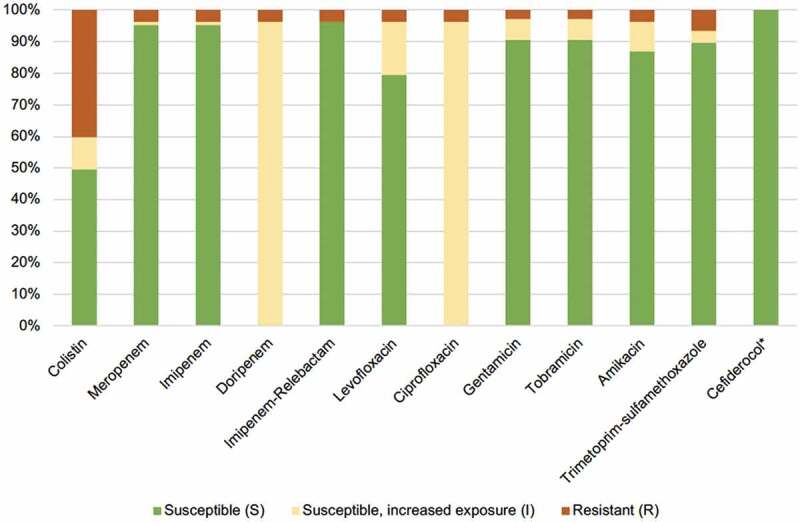
Etest of antibiotic susceptibility on 107 available *Acinetobacter* isolates. *Susceptibility testing based on disc diffusion

**Figure 4.**
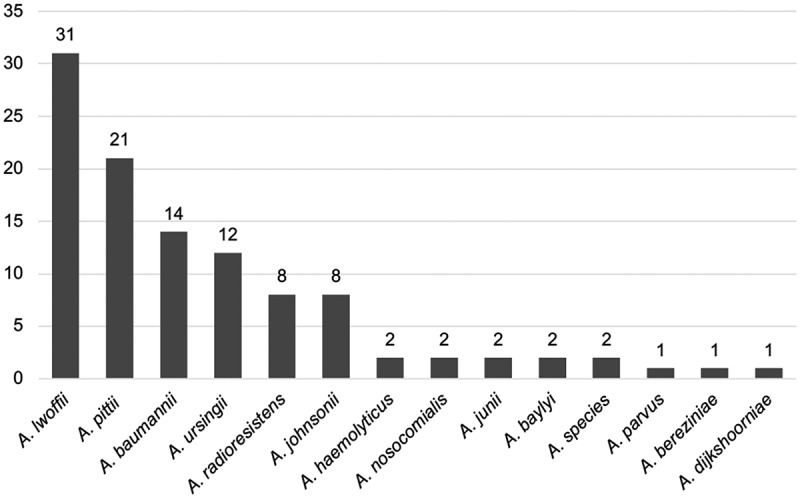
*Acinetobacter* species distribution as determined by MALDI-TOF (matrix-assisted laser desorption/ionization time of flight)

Only five *Acinetobacter* strains out of 169 tested (3%) were carbapenem-resistant, of which four were found in patients hospitalized abroad immediately prior to the *Acinetobacter* bacteraemia. The result of the susceptibility testing is displayed in Figure 3. Additional bacterial species other than *Acinetobacter* were found in 81 (46%) of the bacteraemia episodes. Positive *Acinetobacter* cultures from locations other than blood were reported in 20 (12%) patients. Primary *Acinetobacter* bacteraemia, ie with no other foci of infection identified, was seen in 82 (46%) of cases while 97 (54%) were classified as secondary bacteraemia. In all, 107 isolates were preserved at the Department of Clinical Microbiology and available for MALDI-TOF analysis. *A. lwoffii* was most frequently identified (*n* = 31, 29%) followed by *A. pittii* (*n*= 21, 20%) and *A. baumannii* (*n* = 14, 13%). Other than these, 11 different *Acinetobacter* species were identified (Figure 4).

### The 30-day survival rate was high

As presented in Table 2, patients received empirical antibiotic treatment at bacteraemia onset in 164 of 179 cases (92%). However, only 37 patients (21%) received empirical antibiotics effective against *Acinetobacter*. In total, 133 patients (76%) received antibiotics with proper antibacterial activity against *Acinetobacter* during the course of bacteraemia. The median treatment duration was 10 days (range 1–28) for those effectively treated, either empirically or after adjusting treatment according to the antibiogram. Out of the total 179 cases, 33 patients (18%) received source control treatment, including catheter removal, nephrostomy and wound revision. In 53 cases of bacteraemia (30%), effective antimicrobial treatment targeting *Acinetobacter* were discontinued or not at all initiated, either due to poor prognosis of the patients (unethical to motivate further antibiotic treatment) or the *Acinetobacter* found being considered a contamination.

For all 179 episodes of bacteraemia, all-cause mortality rates were 16% (*n* = 28), 20% (*n* = 36) and 30% (*n* = 54) at 30-days, 90-days and at 1-year, respectively.

### Analyses of putative risk factors for 30-day mortality

In an initial univariate complete case analysis, both Charlson comorbidity index, baseline functional capacity, age, all blood cultures positive for *Acinetobacter* and national/pediatric early warning score (NEWS2/PEWS) were significantly associated with 30-day mortality (Table S1). In the full multivariate model including six variables and multiple imputation for missing data (n = 176), all blood cultures positive had the strongest association with 30-day mortality (OR 5.0, 95% CI: 1.8 to 13.7, *p*= 0.002), baseline functional capacity the second strongest (1–4 points, OR 2.0, 95% CI: 1.2 to 3.4, *p*= 0.010) and finally, correct empiric antibiotics (when the culture was obtained) was also associated with an increased risk for death (OR 3.5 95% CI: 1.0 to 11.8, *p*= 0.045). The final full model showed good discriminatory abilities with an area under the receiver operating curve (AUROC) of 0.86 (95% CI: 0.79 to 0.93) but quite low model fit with an R^2^ of 23.2%. A complete case (CC) analysis (n = 148) shifted the effect estimates for blood culture positivity and baseline functional capacity somewhat, but the main difference was that correct empiric antibiotics and Charlson comorbidity index switched places for which variable had a *p* value below 0.05. The result of the multivariate regression analysis is detailed in Table 3.

**Table 3.** Multivariable logistic regression model with 30-day all-cause mortality as the outcome variable

	Multiple regression model					
Variable	MI *n*= 176	MI	MI	CC *n*= 148	CC	CC
	t-value	OR 95% CI	*p-*value	z value	OR 95% CI	*p-*value
All blood cultures positive *for Acinetobacter*, yes/no	3.10	4.96 (1.80–13.68)	**0.002**	3.24	6.53 (2.10–20.3)	**0.001**
Baseline functional capacity, points (0–4)	2.57	1.99 (1.18–3.37)	**0.010**	2.32	1.90 (1.11–3.26)	**0.020**
Received empirical antibiotics effective against *Acinetobacter*, yes/no	2.00	3.48 (1.03–11.78)	**0.045**	1.91	3.80 (0.97–14.9)	0.056
Charlson comorbidity score, points (0–15)	1.67	1.20 (0.97–1.50)	0.094	2.80	1.43 (1.11–1.84)	**0.005**
NEWS2/PEWS, points (0–12)	1.62	1.14 (0.97–1.34)	0.105	1.80	1.18 (0.99–1.40)	0.072
Age	1.16	1.02 (0.99–1.05)	0.245	−0.19	1.00 (0.97–1.03)	0.853
	Tot MI	95% CI		Tot CC	95% CI	
Model fit, pseudo R^2^	23.2%	-		29.3%	-	
Discrimination, area under ROC	0.86	0.79–0.93		0.86	0.77–0.94	

Results shown for all included variables and both MI and CC analyses. *p-*values below 0.05 were regarded as statistically significant and are written in bold.

MI: Multiple imputation, CC: Complete case, NEWS2/PEWS: National/Pediatric early warning score, ROC = receiver operation characteristic.

## Discussion

The aim of this observational study was to report a detailed account of the epidemiology and outcome of *Acinetobacter* bacteraemia over 10 years in South Sweden. We found a low 30-day all-cause mortality of 16%, a high rate of community-acquired infections and only 3% of the *Acinetobacter* strains were carbapenem-resistant.

The incidence of invasive *Acinetobacter* in our study remained relatively stable throughout the 10 years studied. This is encouraging given the increasingly problematic role of *Acinetobacter* globally and the continuous increase in international travel seen during the last decade [12,13]. Our paper indicates that invasive *Acinetobacter* infections can be kept at a constant level with the use of proper surveillance, infection prevention and control and restrictive carbapenem usage.

The 30-day all-cause mortality of 16% found in this study is low compared to rates of 24–84% reported in previous studies [14–17]. The reason for the low mortality rate could have several explanations. It could be related to the low rate of carbapenem-resistance (3%) found in this study, compared to the carbapenem-resistance rate of 32% of invasive *Acinetobacter* isolates in Europe 2018 [5]. Multiple other studies have linked carbapenem resistance (or multi-drug resistance) to 30-day mortality [3,16]. In this study, only five carbapenem resistant strains were found, why such a comparison could not be performed. In Sweden, the usage of carbapenems in hospital settings is restricted and effective infection prevention measures are since long established to prevent nosocomial transmission of resistant bacteria. Furthermore, mortality could be related to *Acinetobacter* species. We found a predominance of *A. lwoffii* and *A. pittii*, and only a minority of strains were *A. baumannii*. Previous studies found appropriate antibiotic treatment associated with survival in infections caused by *A. baumannii*, but not by the less virulent *A. nosocomialis* [18,19].

The low mortality also raises the question whether *Acinetobacter*, in some cases, might constitute contamination of blood cultures. A mere 21% of patients received empiric antibiotic treatment effective against *Acinetobacter*, and 26% of patients never received adequate antibiotics during the course of bacteraemia. In spite of this, and in spite of other studies recognizing early administration of active antibiotics as a particularly important factor determining clinical outcome in *Acinetobacter* infections, the survival rate was high [3,15,16,20]. In fact, the multivariate logistic regression model found a positive association between empirical effective treatment and 30-day mortality. Previous studies support the hypothesis that *Acinetobacter* might contaminate blood cultures [21–23]. In our multivariable survival model, *Acinetobacter* growth in all blood cultures drawn at the day of bacteraemia onset had the strongest association with 30-day mortality, indicating that several positive blood cultures may be a marker of actual infection. Furthermore, almost half (46%) of the patients in this study had additional species of bacteria in their blood cultures beside *Acinetobacter*, which could indicate contamination. Many clinicians refrained from antibiotic treatment on the suspicion of contamination rather than infection. *Acinetobacter* being a contamination could also explain the low rates of ICU-treated patients, as well as the high rate of community acquired infection (78%); other studies primarily regard *Acinetobacter* as a nosocomial pathogen [24]. However, if the additional microbes also found in the blood culture were for instance other gram-negative rods or *Staphylococcus aureus*, this would not indicate contamination but rather a mixed infection. To what extent *Acinetobacter* bacteraemia constitute a contamination should preferably be studied in future prospective studies.

A strength of this study is the long inclusion time, the population-based nature and few exclusion criteria with few excluded patients and contemporary statistical methods. Limitations include the retrospective nature of the study, resulting in a risk for introducing selection bias. We used putative risk factors in our regression analyses believed to be most clinically relevant. This introduces the risk that other important variables associated with 30-day mortality were overseen. The sample size in our study is relatively small which limits the statistical power of the study. Therefore, regression modelling results should be interpreted with caution, including the association between empirical antibiotic treatment and mortality. Furthermore, since many of the standardized variables were collected from unstandardized written medical record, there is a risk of information bias. In addition, some variables have rather large proportions of missing data, although this was handled using multiple imputation. Furthermore, susceptibility testing and characterization to subspecies could only be performed in 107 of 179 strains, as the remaining were lost over time. In addition, we used Etest for testing colistin susceptibility, rather than the broth microdilution method recommended by EUCAST. This could have rendered a falsely high rate of isolates exhibiting colistin resistance and susceptibility, increased exposure.

## Conclusions

In this retrospective cohort study of a decade of *Acinetobacter* bacteraemia in the south of Sweden, we found a high degree of community-acquired infection, a low rate of carbapenem resistance and low 30-day mortality, compared to previous international studies. This may reflect a high rate of microbiological contamination rather than clinical infection.

## Supplementary Material

Supplemental Material
