# Supplementary material for: Low 30-day mortality and low carbapenem-resistance in a decade of Acinetobacter bacteraemia in South Sweden
Source: Infect Ecol Epidemiol. 2021 Dec 10;12(1):2009324. doi: 10.1080/20008686.2021.2009324 (PMC8667949; doi:10.1080/20008686.2021.2009324)
Supplement: Supplemental Material [file ZIEE_A_2009324_SM9167.docx]

Supplementary data

**Table S1.** Univariate logistic regression model with 30-day all-cause mortality as the outcome variable.

|  | *n* | Univariate model | | |
| --- | --- | --- | --- | --- |
| Variable |  | **LR chi^2^** | **OR 95% CI** | ***p*-value** |
| Charlson comorbidity score, points (0-15) | 176 | 16.76 | 1.294 (1.1333-1.477) | <0.001 |
| Baseline functional capacity, points (0-4) | 156 | 14.89 | 2.257 (1.476-3.453) | <0.001 |
| Age, years (0-94) | 176 | 10.71 | 1.029 (1.008-1.050) | 0.005 |
| All blood cultures positive *for Acinetobacter,* yes/no | 170 | 8.51 | 3.500 (1.467-8.351) | 0.005 |
| NEWS2/PEWS, points (0-12) | 167 | 4.16 | 1.143 (1.004-1.300) | 0.043 |
| Received empirical antibiotics effective against *Acinetobacter,* yes/no | 174 | 1.00 | 1.614 (0.646-4.030) | 0.305 |

All variables of interest are analysed univariately against the outcome (all-cause mortality within 30 days) on their original scale using logistic regression and complete case analysis. Variables are sorted after strength of the association with the outcome based on LR chi^2^.

LR: likelihood ratio. NEWS2/PEWS: National/Pediatric early warning score, OR: Odds ratio
